# Information Traceability Model for the Grain and Oil Food Supply Chain Based on Trusted Identification and Trusted Blockchain

**DOI:** 10.3390/ijerph19116594

**Published:** 2022-05-28

**Authors:** Xin Zhang, Yue Li, Xiangzhen Peng, Zhiyao Zhao, Jiaqi Han, Jiping Xu

**Affiliations:** 1 School of Artificial Intelligence, Beijing Technology and Business University, Beijing 100048, China; zhangxin@btbu.edu.cn (X.Z.); 15810428206@163.com (Y.L.); 2030602060@st.btbu.edu.cn (X.P.); zhaozy@btbu.edu.cn (Z.Z.); 18612930577@163.com (J.H.); 2 Beijing Key Laboratory of Big Data Technology for Food Safety, Beijing Technology and Business University, Beijing 100048, China; 3 Key Laboratory of Internet and Big Data for China Light Industry, Beijing Technology and Business University, Beijing 100048, China

**Keywords:** grain and oil food, information traceability model, trusted blockchain, trusted identification, Hyperledger Fabric, food safety

## Abstract

The grain and oil food supply chain has a complex structure, long turnover cycles, and many stakeholders, so it is challenging to maintain the security of this supply chain. A reliable traceability system for the whole grain and oil food supply chain will help to improve the quality and safety of these products, thus enhancing people’s living standards. Driven by the trusted blockchain and trusted identity concepts, this paper constructs an information traceability model for the whole grain and oil food supply chain, and it describes how contract implementation and example verification are performed. First, an information traceability model framework of the whole grain and oil food supply chain is established based on the survey and analysis of the grain and oil food supply chain. Second, trusted identification, blockchain master–slave multi-chain storage, and trusted traceability mechanisms are designed. The trusted identification mechanism is used to track the data information of the whole grain and oil food supply chain. The blockchain master–slave multi-chain storage solves the problem of miscellaneous information caused by many links in the whole grain and oil supply chain, while the credible traceability mechanism ensures the credibility of information collection, storage, and transmission. Finally, based on the data flow, the model operation process is analyzed. Using the information traceability model, the grain and oil food trusted traceability system is designed and developed with the Hyperledger Fabric open-source framework, and a case study is conducted to verify the system. The results show that the model and system constructed in this study solve the problems of low data security and poor sharing, which exist widely in the traditional traceability mechanism, and enable the trusted uplink, storage, processing, and traceability of multi-source heterogeneous information in the lifecycle of the whole grain and oil food supply chain. The proposed system improves the granularity and accuracy of grain and oil food traceability, and provides support for the strategic security of grain stock.

## 1. Introduction

Grain and oil food, including raw grain, finished grain (grain after processing) and edible oil, are the necessities of national life [1]. In 2021, China’s grain output will hit a record high again, stabilizing at more than 1.3 trillion catties for seven consecutive years, and grain and oil food occupy the dining table of the Chinese people almost every day. Grains serve as an important food source of nutrients such as B vitamins, minerals, and dietary fiber, according to relevant literature research, and whole grains can help prevent cardiovascular and metabolic diseases [2]. The type of oily food has a certain influence on the control of fatty acid intake required by the human body [3]. Ensuring the safety of grain and oil food supply is of great significance to the life and health of the people and the harmonious development of society. The grain and oil food supply chain includes multiple participating links such as cultivation, processing, warehousing, logistics, and retailer [4]. The supply chain cycle is long, and there are many factors that threaten the safety of grain and oil food in each link. In the grain and oil food supply chain, there is excessive use of pesticides and chemical fertilizers and unclear processing additives, which lead to quality safety problems in the finished grain and oil food; problems such as lack of warehousing and logistics information and counterfeiting of retailers lead to the inability of traceability information to flow, so it is impossible to trace the problem, which poses a serious threat to public health [5]. Therefore, it is urgent to establish a safe and effective reliable traceability platform for the whole supply chain of grain and oil food, so as to improve the transparency and reliability of participation in all links of the whole supply chain, which can effectively protect the rights and interests of consumers and improve the efficiency of supervision [6]. In addition, while ensuring the sustainable development of the quality and safety of the entire supply chain of grain and oil food, it also ensures the health of the people [7]. The traditional grain and oil food traceability systems are mostly centralized models [8]. Some core enterprises collect and manage the data of the supply chain in a centralized manner, and store the data in a centralized server. However, the centralized management model has low security and is vulnerable to tampering. Although some enterprises use two-dimensional code, RFID and other identification technologies to track data in the supply chain, they cannot guarantee the transparency and reliability of data [9]. Therefore, the traditional traceability model has not eliminated consumers’ doubts about the safety and reliability of grain and oil food products [10].

Blockchain is a decentralized distributed ledger technology, which ensures that the data on the chain cannot be tampered with and forged through mechanisms such as encryption and consensus. In recent years, the application of blockchain has gradually penetrated from the encrypted digital currency represented by Bitcoin to various fields such as finance, medical care, government affairs, justice, public welfare, commodity anti-counterfeiting and food safety [11]. The decentralized architecture of the blockchain and the technical characteristics that data cannot be tampered with and forged provide an effective solution for the trusted traceability of the entire supply chain of grain and oil food [12].

Based on trusted blockchain and trusted identification, this paper designs a set of trusted traceability models for the entire supply chain of grain and oil food, providing information on the entire process from the production link to the hands of consumers to ensure data security and reliability. On the basis of this model, a trusted identification mechanism is designed, and the master–slave multi-chain blockchain uses a storage mechanism and a trusted traceability mechanism to verify the model. These three mechanisms are supported by technologies such as industrial Internet identification, QR codes, and blockchain. In the event of a food safety accident, the food traceability system can quickly, accurately and efficiently locate the link where the problem occurred, find the root cause of the problem, track the whereabouts of the product in question, identify the responsible enterprise and hold them accountable, reduce the adverse effects caused by the food safety accident, and allow the majority of consumers to have a clearer understanding of the data information of the entire supply chain of grain and oil food from farmland to dining table, which can effectively solve the problem of criminals tampering with data and information in the supply chain. Moreover, since the model mostly relies on automated equipment for information collection and processing, it solves the problem of too many participants in the supply chain to a certain extent. It is of great significance to solve food safety problems, and can effectively improve human health problems caused by food safety problems.

## 2. Research Methodology

### 2.1. Trusted Blockchain

Blockchain is a distributed ledger technology supported by computer technologies such as distributed data storage [13,14], peer-to-peer network transmission, encryption algorithms, and consensus mechanisms, and is essentially a distributed database [15]. Due to the continuous improvement of the security performance of the blockchain in recent years, the blockchain has been called a trusted blockchain by a large number of researchers. Each data block in a trusted blockchain includes a block header and a block body. The block header consists of the current block version, the current hash value, the previous block hash value, the timestamp and a random number [16]. The block body consists of the data structure that contains the verified records of all transactions generated during the block creation process. This chain-like data structure encrypted by an encryption algorithm ensures that the data in the trusted blockchain cannot be tampered with, thereby ensuring data security in all links in the entire supply chain. At the same time, through the consensus mechanism, all nodes in the trusted blockchain network participate in the data authentication process [17]. Trusted blockchain ensures the consistency and authenticity of data on the chain, and solves the problems of “information islands” and data opacity in traditional centralized systems [18]. When a smart contract deployed in a trusted blockchain network reaches a certain trigger condition, it can automatically execute the preset program code jointly verified by all nodes. Since the execution process and results are open and transparent to all nodes in the entire network [19], trusted blockchain can effectively solve the trust problem between all nodes [20]. Therefore, it can help increase the trust between the cultivation, processing, warehousing, logistics, retailer and final consumption of the entire supply chain, and ensure the safety and reliability of traceable data throughout the grain and oil food supply chain.

### 2.2. Trusted Identification

As an important means of carrying information directly and effectively, identification technology is self-evident in the importance of information tracking and traceability in the whole supply chain of grain and oil food [21,22]. How to effectively track and trace the data generated by the cultivation, processing, warehousing, logistics, retailer and other participating links in the whole grain and oil food supply chain and ensure the real-time, safety and reliability of the data in the traceability system has become a research hotspot in recent years [23]. Currently, widely used identification technologies include barcode, RFID, NFC, EPC and other IoT-based electronic identification technologies [24]. Barcode technology includes one-dimensional barcodes and two-dimensional barcodes [25]. One-dimensional barcodes are limited by their low storage capacity and are mostly used for simple product numbering in combination with traditional centralized databases; two-dimensional barcodes have high information density, large capacity, strong fault tolerance, low cost, convenient and flexible use [26]. It can be better applied to the traceability of the whole supply chain of grain and oil food based on blockchain [27]. As a non-contact radio frequency identification technology, radio frequency identification (RFID) technology is used to read and write RFID tags attached to products through a reader [28], and it is being utilized in more and more food products. However, due to the cost and security of RFID tags, it still faces challenges before large-scale commercial use in the future [29]. The trusted identification in this paper is to generate the unique identification code of the product through the industrial Internet identification technology, and store the identification code into the two-dimensional code through the two-dimensional code technology, so that the information circulation of the product is more secure and reliable.

### 2.3. Research Progress

In recent years, due to the advent of the blockchain 2.0 era, many scholars have used blockchain technology to combine with the food supply chain traceability system to design safe, reliable and efficient traceability solutions. At the same time, based on barcodes and RFID, the research on the traceability system of food supply chain with other technologies has a long history. Among them, some frontier studies on food or crop traceability based on blockchain or identification technology are shown in Table 1.

Salah et al. proposed a soybean supply chain traceability solution based on the Ethereum smart contract technology and the interstellar file system, which effectively improved the information transparency and traceability of the soybean supply chain traceability system [30]. Tao et al. proposed a comprehensive evaluation model based on blockchain smart contracts, which can automatically detect and pre-warn food quality in the food industry chain, improving the safety and credibility of the food industry chain [31]. Nuno et al. provided a general M2M secure transaction scheme through blockchain and smart contract technology, and proposed another method for the food security transaction problem [32]. Lin et al. proposed a food safety traceability prototype system based on blockchain and EPC information services, and avoided the problems of data tampering and sensitive information leakage that may occur in the process of information interaction through the Ethereum smart contract [33]. Varavallo et al. proposed a green blockchain traceability platform based on the Internet of Things and blockchain technology, which provides an environmentally friendly and low-cost traceability solution for food traceability [34].

Wang et al. proposed a food traceability system based on blockchain + RFID technology. The system includes a creatively designed data structure supporting blockchain in the RFID tag, which improves the safety and reliability of product data [35]. Caro et al. proposed AgriBlockIoT, a traceability solution for agricultural products based on the Internet of Things and blockchain, and supports the expansion of IoT sensor devices, ensuring data transparency, fault tolerance, and non-tampering [36]. TSANG et al. proposed a food traceability system based on blockchain and IoT technology, which provides support for the quality traceability management of food throughout its life cycle [37]. L. Sun et al. proposed a smart agricultural technology based on the Internet of Things, which provided support for promoting the development of modern agriculture, saving agricultural production costs, and increasing production and sales [38]. M.S et al. [39] proposed a source-aware traceability framework for IoT-based supply chain systems, the ProChain framework, which is essentially a traceability-aware solution designed for IoT-based supply chain systems, the scheme provides a new line of traceability for food traceability. Yang et al. proposed a blockchain and industrial IoT edge data sharing framework, which can be used for reference to improve the security and trustworthy sharing of grain and oil food traceability information [40].

In recent years, the development of circular economy and agriculture has gradually become one of the hot spots. Duque-Acevedo et al. conducted in-depth research on circular economy-related literature and agriculture-related literature, and concluded that agriculture is the preferred source of circular economy production models, reflecting the importance of agriculture to circular economy [41]. Meng, XY et al. believe that agriculture is one of the most effective ways to implement sustainable development, and divide the agricultural circular economy in Heilongjiang Province, China through fuzzy min-max neural network with fuzzy lattice inclusion measure [42]. Rodias et al. concluded from a survey of literature that the implementation of circular economy practices in resource-consuming agricultural systems is essential for reducing the environmental ramifications of the currently linear systems [43].

To sum up, the adoption of IoT technologies based on blockchain and identification has become a hot spot in the research on information traceability of the whole grain and oil food supply chain [44]. However, in terms of data storage, existing research cannot solve the problem of large and complex data in grain and oil food or agricultural supply chain; in terms of identification, there is still a lack of a reliable and safe identification method. So, the research of this paper will use the trusted blockchain and trusted identification to trace the information of the whole supply chain of grain and oil food. The characteristics of blockchain decentralization, high transparency, and data that cannot be tampered with and forged, combined with barcode and other identification technologies to track and trace data in all aspects of the grain and oil food supply chain, including production, processing, warehousing, transportation, and sales, can effectively improve grain and oil food. Transparency and reliability of information in the entire food supply chain. It is of great significance for the formation of a safe, credible and efficient grain and oil food supply system.

## 3. Materials and Methods

### 3.1. Information Traceability Model of the Whole Supply Chain of Grain and Oil Food

On the basis of the research on trusted blockchain and trusted identification and the analysis of the latest research results, this section firstly analyzes the information of the whole supply chain of grain and oil food. The information traceability model framework of the whole supply chain of grain and oil food is developed.

#### 3.1.1. Analysis the Supply Chain of Grain and Oil Food

**Figure 1:**
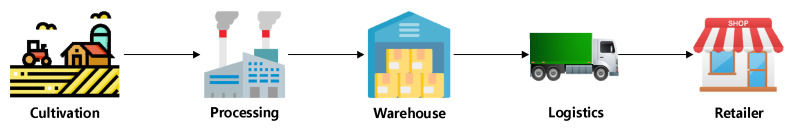
Typical links in the whole supply chain of grain and oil food.

The grain and oil food supply chain has a long cycle and many participants, and almost every link is nested with other links. For example, the breeding link includes warehousing, logistics, and even simple rough processing, processing, and retailing. It is also inseparable from the basic logistics, warehouse and other links. Its complexity and uncontrollable risk factors determine the low efficiency and reliability of traditional traceability solutions. Based on the reference of agricultural supply chain articles [45], this section analyzes the participating links of the entire grain and oil food supply chain, and extracts the five most typical links in the entire grain and oil food supply chain, namely, cultivation, processing, warehouse, logistics and retailer. The whole grain and oil food supply chain abstract model composed of these five typical links has high adaptability and flexibility to different grain and oil food supply chains, can be expanded and adapted according to the actual situation of different grain and oil food supply chains. The typical link of the whole supply chain of grain and oil food proposed in this paper is shown in Figure 1, and the follow-up research work will also be carried out based on this typical link.

The tracking and tracing information of typical links in the whole grain and oil food supply chain mainly includes the following: the relevant information of the grain and oil food planting and harvesting process in the cultivation link, the detailed information of the rough processing, fine processing and other stages of the product in the processing link, as well as the batch, serial number and quality of the product, inspection and other information, product storage warehouse number, entry and exit time and other information recorded in the warehousing process, information such as logistics vehicles, logistics personnel, and logistics routes in the logistics process, and retailer locations, personnel, dealers, and product shelf times in the retailer process, and other information. The information collected by each participating link in the whole grain and oil food supply chain is verified and entered into the traceability system based on the trusted blockchain, so as to efficiently track and trace the information of the entire supply chain link of a certain batch of products.

#### 3.1.2. Overall Framework of the Model

**Figure 2:**
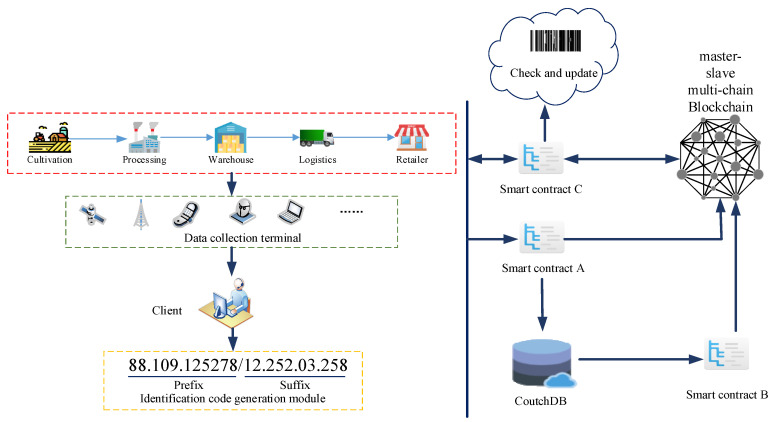
Schematic diagram of the model frame.

Aiming at the above-mentioned typical links of the whole grain and oil food supply chain abstracted, combined with the actual operation of enterprises on the offline supply chain, this paper designs a framework of information traceability model of the whole grain and oil food supply chain based on trusted blockchain and trusted logo. The framework consists of a collection end, a client and a storage module. The collection end includes five typical supply chain links of cultivation, processing, warehouse, logistics, retailer and the corresponding collection equipment; the client includes the enterprise users, regulatory authorities and consumers corresponding to the five typical links. Data information is verified and queried. In addition, each client is embedded with a logo generation module; the storage module is composed of a blockchain and a cloud database. Each part of the model is directly called by the customized smart contract to achieve interconnection. This model is based on the principle of blockchain, combined with smart contract technology and identification analysis technology, and designs a credible traceability model for grain and oil food. The schematic diagram of the model framework is shown in Figure 2.

Based on the principle of blockchain, this model combines smart contract technology and identification analysis technology to design a reliable traceability model for grain and oil food. On the basis of the overall model, this study designs three operating mechanisms that conform to the model: trusted identification mechanism based on trusted blockchain, blockchain master–slave multi-chain storage mechanism, and trusted traceability mechanism.

The trusted identification mechanism is constructed through “trusted blockchain + trusted identification”. After the data information of the supply chain is collected through the Internet of things equipment, the technicians use the industrial Internet identification technology to create a unique identification code for it, and then store the identification code in the QR code through the QR code technology to perform the next operation; the master–slave multi-chain storage mechanism of the blockchain aims to solve the problem that the data and information of the grain and oil food supply chain are numerous and complicated, which cannot be satisfied by the traditional single chain. The problem of traceability and timeliness is to increase the storage capacity by designing the main chain and the slave chain of the blockchain network. The main chain is the query chain, and the slave chain is the data information storage chain. The main chain only needs to store the data uploaded by the slave chain through the smart contract. For a small amount of information such as abstracts and ID values, when the user needs to trace the source of the query information, he can query the smart contract again through the main chain to call the required data information from the slave chain; the trusted traceability mechanism is stored in the trusted identifier and the master–slave multi-chain. On the basis of the mechanism, all links of the grain and oil food supply chain are integrated into it to form a safe and reliable credible traceability mechanism. After verification, the three mechanisms can effectively fit into the grain and oil food supply chain to form credible traceability, thereby improving the quality and safety of grain and oil food.

### 3.2. Model Mechanism Design

Based on the framework of the trusted traceability model of grain and oil food, this section designs the trusted identification mechanism, the blockchain master–slave multi-chain storage mechanism, and the trusted traceability mechanism. The trusted identification mechanism realizes the efficient and credible traceability of grain and oil information; the master–slave multi-chain storage mechanism of the blockchain solves the complicated data storage of grain and oil food, and shares the storage pressure of the data on the blockchain; the trusted traceability mechanism realizes It ensures the credible flow of information in the whole supply chain of grain and oil food between each link, and ensures the credibility of traceability information from collection to retailer. Additionally, the custom-designed smart contract realizes the trusted identification mechanism, the blockchain master–slave multi-chain storage mechanism, and the internal operation logic encapsulation of the trusted traceability mechanism to ensure the smooth operation of the model.

#### 3.2.1. Trusted Identification Mechanism

The trusted identification mechanism based on “trusted blockchain + trusted identification” is used to track data information throughout the supply chain. The mechanism includes the specific appearance of the identification generation module embedded in the client system, the specific process of generating the identification, and the design of the identification.

The trusted identification mechanism firstly verifies and adjusts relevant data in each link of the grain and oil food supply chain, records it in the blockchain system through smart contracts, and automatically executes it after collecting data through relevant equipment. In the cultivation process of the supply chain, the cultivation information of food crops is first entered into the system by the relevant personnel through the client, and then verified and standardized through the smart contract, and then enters the trusted blockchain network, and the relevant information in the supply chain is verified through the feedback the identity information for the smart contract.

**Figure 3:**
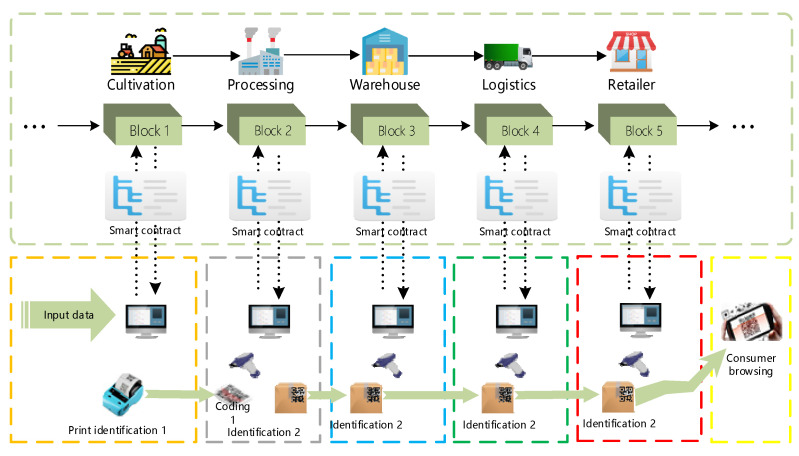
Trusted identity mechanism based on blockchain.

The mechanism prints the corresponding identification code during the processing for the identification of subsequent products. The generated identification code is verified by a smart contract embedded in the blockchain system, and the identity mechanism can be made credible using the smart contract technology of the blockchain. In the processing link, since the application of the identification code to query the data of this link will reduce the timeliness of the actual work, the current more convenient two-dimensional code technology is used to store the information in the identification code and the processing link information into the two-dimensional code, so as to facilitate the work. The staff obtains the product data information by querying the barcode through the QR code scanner. At the same time, relevant product information is added to the system, and after the verification and standardization of the smart contract, it enters the blockchain network, thereby realizing the credibility of the information identification of the two-dimensional code including the identification code. In the final stage of product cultivation and processing, as the product circulates in the supply chain, the product’s QR code is printed on the product’s outer packaging as a basis for subsequent identification and verification. The operating principles and processes of the product identification mechanism in warehousing, logistics, and retailer are similar, except that there is no new entity QR code generation, only the content in the blockchain system is updated, so it will not be described here. Figure 3 shows the composition and working principle of the trusted identification mechanism based on trusted blockchain. The identification code technology realizes the transparency, safety and credibility of the information flow of grain and oil food in the supply chain by combining with the blockchain smart contract technology.

**Figure 4:**
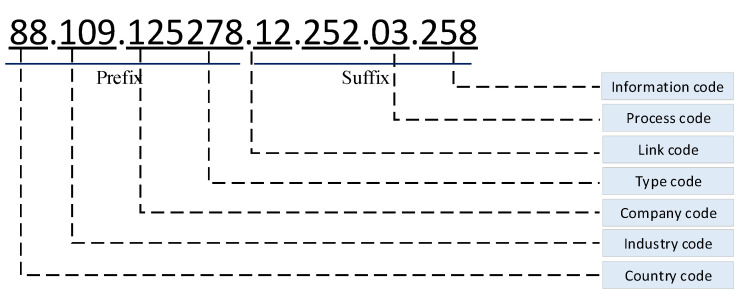
Coding diagram.

In the process of printing the identification code, the corresponding identification code is generated by the embedded identification code generation module according to the uploaded information. The specific code is shown in Figure 4, which includes the header and the suffix. The prefix includes the type code and company code, industry code, country code. The suffix includes information code, process code, and link code.

#### 3.2.2. Blockchain Master–Slave Multi-Chain Storage Mechanism

**Figure 5:**
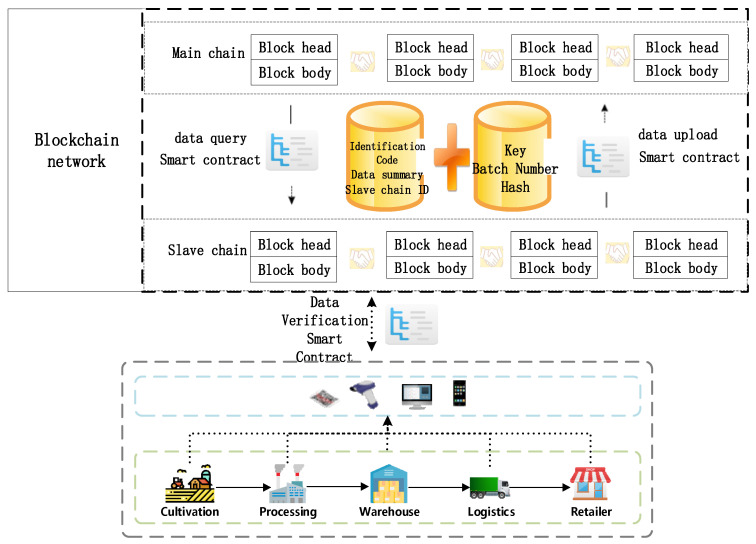
Data storage mechanism based on blockchain master–slave multi-chain.

Because the whole supply chain of grain and oil food is the most numerous and complicated in the food supply chain, and the supply chain involves many links, the amount of data that needs to be collected, recorded and uploaded from cultivation to retailer is huge and complex. If all the data of the link is stored on the blockchain, there will be problems such as high cost, heavy burden, and low efficiency. Therefore, this mechanism designs a storage mechanism based on the blockchain master–slave multi-chain architecture, that is, the products are stored in the blockchain master–slave chain architecture through data verification smart contracts in the supply chain, and the database uses the Hyperledger Fabric blockchain network. With its own CoutchDB database [46], the uploaded data will be broadcast in the blockchain network of the slave chain. The consensus nodes on the chain will package the data into blocks and send them to each node. After each node passes the verification, the data will be stored in the ledger. At the same time, the consensus node in the slave chain will upload the slave chain ID, slave chain data key value, identification code, data digest, product batch number and hash value to the main chain through the relevant smart contract, and pass the hash value. The method of locking will interconnect from the chain to the main chain. In order to protect data security, enterprises in all links of the food supply chain will work together to maintain the security of the main chain, and relevant regulatory agencies and corporate departments will maintain the security of the secondary chain to prevent lawbreakers from destroying data. The slave chain is responsible for storing the data information of each link in the supply chain, and the master chain provides the traceability information query function. After the mechanism is authenticated, it will become a non-forkable main chain block. If you want to change the main chain block, the cost is huge and cannot be realized. At the same time, the master–slave chain intends to adopt a different consensus mechanism to improve the timeliness of the entire system. The main chain adopts PBFT (Practical Byzantine Fault Tolerance), and the slave chain adopts PoW (Proof of Work). Figure 5 shows the storage mechanism of the whole supply chain of grain and oil food based on the master–slave multi-chain blockchain. This storage mechanism means storage on the blockchain. Specifically, the data information is stored in the database CoutchDB in Hyperledger Fabric. Before uploading to the blockchain network database, the information needs to be verified by the data verification smart contract before uploading.

The data uploaded to the blockchain in each link of the grain and oil food supply chain must follow the constraints of the smart contract, and only the data that meets the requirements of the smart contract can be uploaded successfully. At the same time, the smart contract will unify the format of the data uploaded by different nodes in the supply chain, and then verify the data through digital signature technology and upload and store it in the CoutchDB database. The specific process of uploading a large amount of data generated by each link of the entire supply chain to the secondary chain of the blockchain is as follows: a hash operation is performed on a large amount of basic data of the product to obtain a data abstract, and the system generates public and private keys through asymmetric encryption technology. The data digest is encrypted by the private key, as shown in Formula (1), where skp is the private key, D is the standardized data, and Ds is the data digest. After the encrypted data digest is uploaded to the slave chain of the blockchain together with the basic data as the signature value, the received encrypted data digest (signature value) will be decrypted using the public key, as shown in Formula (2), where pkp is the public key, and RDS is the CoutchDB database. The data digest is encrypted by skp. After the encrypted data digest is uploaded to the CoutchDB database as a signature value together with the basic data, the CoutchDB database will decrypt the received encrypted data digest (signature value) through pkp. If the comparison results are consistent, it proves that the data is true and reliable and has not been tampered with, and a large amount of basic data is successfully stored in the CoutchDB database; if the results are inconsistent, it means that the data has been tampered with, does not pass the verification and cannot be stored in the database. Through digital signature technology, the data stored in the CoutchDB database by each enterprise node is valid and credible, so the authenticity of a large amount of basic data in the CoutchDB database is guaranteed. At the same time, since only the data abstracts generated by hashing the basic data and product label data in the CoutchDB database are stored in the blockchain network, the efficiency and performance of the information traceability system for the entire grain and oil food supply chain are guaranteed.

(1) $$Send(D)=file{\left(D,Ds\right)}_{skp}$$

(2) $$Accept(Ds)=file(RDS{\left(Ds\right)}_{pkp})$$

Based on the master–slave multi-chain storage mechanism, this research designs two smart contracts, SDSC (Storage Data smart contract) and VDSC (Verify Data smart contract) to encapsulate the logic rules of the master–slave multi-chain storage mechanism. SDSC is shown in Algorithm A1. VDSC is as shown in Algorithm A2. Among them, H(i) is the user’s unique identity hash, Data is the collected initial data, D is the standardized data, Ds is the data summary, RDS is the CoutchDB database, and R is the interaction record. For Algorithm A1: SDSC and Algorithm A2: VDSC, see Appendix A for details.

The storage mechanism of the master–slave multi-chain of the blockchain is achieved through the digital encryption technology of the trusted blockchain, and the data stored in the CoutchDB database by each enterprise node is effective and reliable; thus, this ensures the authenticity of a large amount of data in the CoutchDB database. At the same time, since the blockchain network only stores the data summaries generated by the hashing of basic data and product label data in the CoutchDB database, efficient supply chain performance is guaranteed.

#### 3.2.3. Trusted Traceability Mechanism

**Figure 6:**
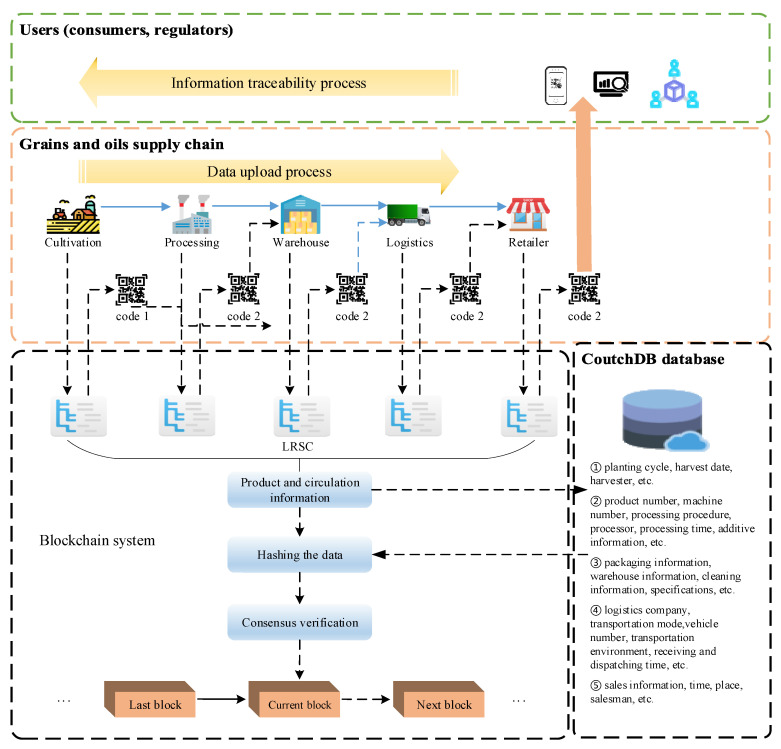
Credible traceability mechanism of the whole supply chain of grain and oil food.

Based on the typical links of the whole grain and oil food supply chain, on the basis of the trusted identification mechanism and the master–slave multi-chain storage mechanism of the blockchain, the trusted traceability mechanism of the whole grain and oil food supply chain information designed in this paper is shown in Figure 6. The information collection, storage and transmission of all links in the whole grain and oil food supply chain realized by the combination of trust blockchain and trusted identification are credible.

The product label information of each link of the grain and oil food supply chain and the basic data in the CoutchDB database are hashed to obtain the hash value, and the independently maintained blockchain node sends the data summary to the blockchain, and then broadcasts it to the entire network. All nodes in the entire network are verified by the consensus process and then deposit the latest data block, resulting in a trusted traceability blockchain for the entire grain and oil food supply chain. End consumers or regulatory agencies scan the QR code of the product through the client or enter the product code of the batch to be queried in the traceability system to trace the entire supply chain information of grain and oil food. The product code is directly located to the specific block header containing the product information. Call out the data of all circulation links of this product for product traceability. By setting different permissions on the QR code, the application of different traceability scenarios such as consumers and supervisors can be realized. The supervisor can scan the QR code of the commodity through the client to realize the traceability of all the information in each link of the whole supply chain of the commodity, which greatly improves the effectiveness of grain and oil food supervision, and at the same time, it can efficiently track and accurately locate the problem link in the event of a grain and oil food safety accident. The rights of consumers are limited to general commodity information and circulation information, and the sensitive information of enterprises in the cultivation and processing process will not be displayed to ordinary consumers, so as to achieve the protection of participants in the process to a certain extent.

At the same time, this mechanism designs the identification resolution smart contract LRSC (identification resolution smart contract) to realize the logical encapsulation of the trusted traceability mechanism, as shown in Algorithm A3. The identification addition information is shown in Equation (3). Among them, code is the identification code, Dc is the information in the identification code, and Dd is the link data. For Algorithm A3: LRSC, see Appendix A for details.

(3) $$Ds=\sum_{i=1}^{n}Ds_{i}$$

The above-mentioned information traceability mechanism of the whole grain and oil food supply chain designed in this section realizes the trusted traceability of the whole grain and oil food supply chain information through the use of trusted blockchain and trusted identification. The model improves the efficiency and cost of the traceability process. The design of the QR code to identify different authorities protects the participants in all aspects of the whole supply chain of grain and oil food, improves the acceptability of the traceability system, and effectively ensures the traceability of information in the whole supply chain of grain and oil food, as well as transparency, credibility and efficiency of regulation.

## 4. Results and Discussion

Based on the trusted traceability model and three mechanisms of the grain and oil food supply chain, this section analyzes the operation process of the grain and oil food supply chain information traceability model, and establishes a grain and oil food trusted traceability system based on Hyperledger Fabric. The system operation has been verified for practicality.

### 4.1. Model Running Process Analysis

**Figure 7:**
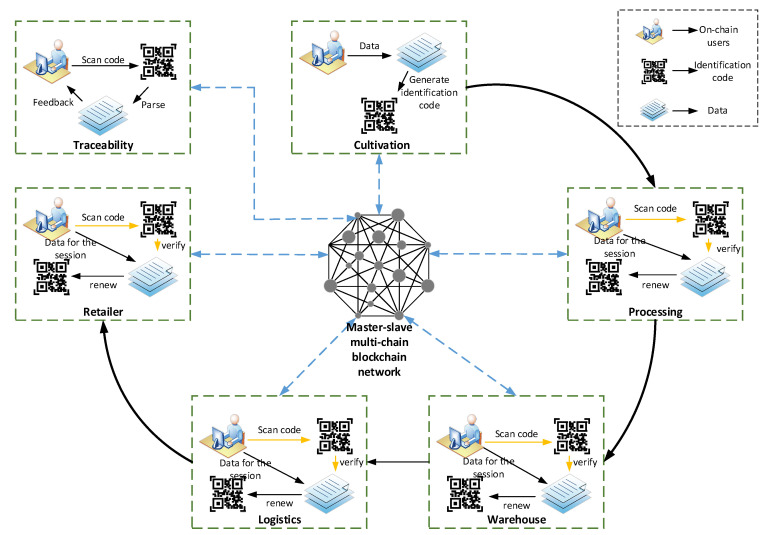
Schematic diagram of the operation process of the system model.

Based on the trusted traceability model of the whole supply chain of grain and oil food, when a user or regulatory department initiates a traceability request through the traceability system, the data flow running through the traceability system can be divided into two processes: data upload and information traceability. The data upload process mainly includes the data upload process of the whole supply chain of grain and oil food and the traceability of the data operation process in the blockchain system. The information traceability process is mainly for the user to initiate a traceability request through the terminal scanning the traceability identification code. The schematic diagram of the running process of the system model is shown in Figure 7.

**Figure 8:**
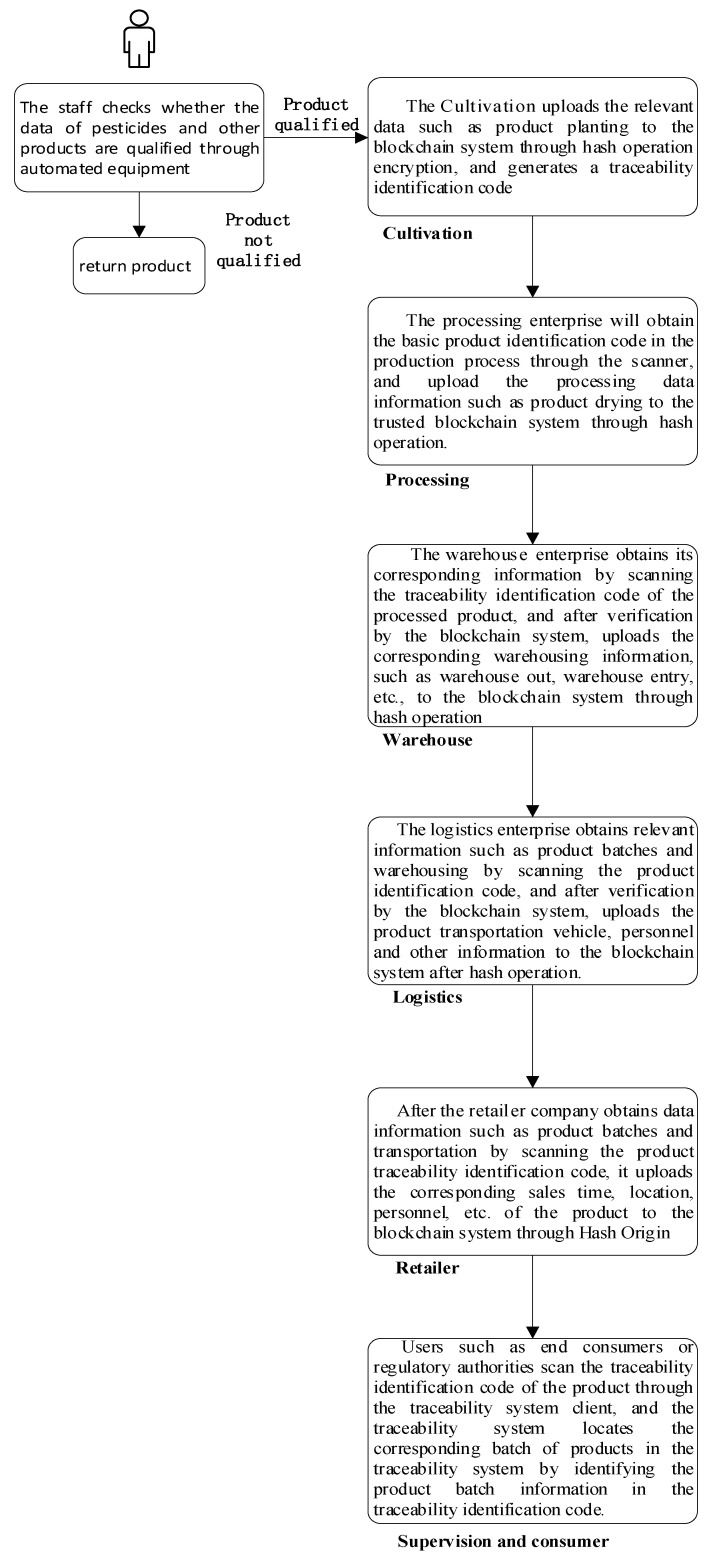
System-relevant data information flow chart.

The specific flow chart of the relevant data in the system is shown in Figure 8.

### 4.2. Systematic Realization

**Figure 9:**
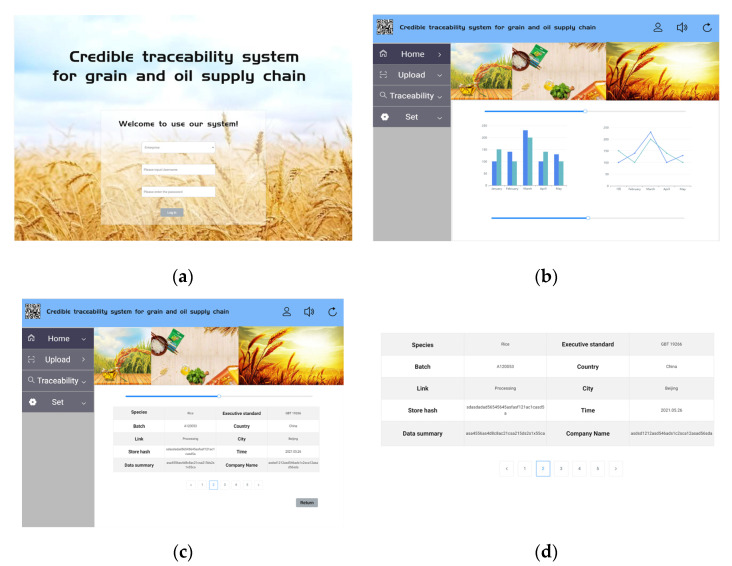
Schematic diagram of the prototype system interface. (**a**) Shows the login interface of the system, the user selects the corresponding category to log in to the system to obtain permissions; (**b**) shows the main interface of the system, the main interface consists of two departments, and the top is the advertisement carousel of the merchants, and the bottom part shows the recent system login times and the number of rice batches that have been attacked recently; (**c**) shows the enterprise uploading relevant data interface, including grain and oil types, batches, links, company name, time, data summary and other information; (**d**) displays the traceability information of the corresponding supply chain link of the product.

Based on the information traceability model of grain and oil food supply chain, this paper builds a prototype system of grain and oil food trusted traceability system. The prototype system used Linux version 16.0.0 and Ubuntu version 20.04.1. On this basis, use the Hyperledger Fabric 2.1 Kaiyuan framework to build the system, in which the docker version is 20.10.7 and the Go language version is 1.17.2. The system adopts the Kafka consensus mechanism. For the 5 classic links and regulatory agencies, 5 organizations, 10 nodes and 3 sorting nodes are set up to build the prototype system. The system interface diagram is shown in Figure 9.

Reliable traceability systems locate specific information about product batches, times and other supply chain links based on the product number of the QR code. Senior personnel such as regulators and administrators can use product QR codes to track the movement of products throughout the supply chain. Ordinary users (such as consumers) can only query basic non-sensitive information about the product.

### 4.3. System Operation Result Analysis

To ensure the credible traceability of the data and information of the whole grain and oil food supply chain, ensure the safety, openness and transparency of the data and information, so as to ensure the health of the general public, it is necessary to collect, transmit and store the information of each link of the whole grain and oil food supply chain for analysis of the safety factors. In terms of data and information collection security, because there are many links in the entire grain and oil food supply chain, including cultivation, processing, warehouse, logistics, retailer and other links, and each link is composed of many parts, resulting in huge and complex data in each link, and for each link the quality of personnel varies, the data collection in the information traceability model of the whole grain and oil food supply chain is inevitably faced with difficulties, which represents a big security risk. For example, in the cultivation process, farmers or cultivation enterprises may not collect timely and accurately information on grain and oil crops, due to the low level of informatization; in the processing, the actual processing procedures, additives and other information of the processing company cannot guarantee its authenticity. The complexity of the information collection process in the entire supply chain determines that no traceability model can guarantee the absolute security of the collected data.

In terms of transmission security, smart contracts are used to determine whether the data meets the requirements during data transmission in the entire grain and oil food supply chain. Only the data collected and uploaded by participants such as current enterprises or farmers meets the preset conditions of smart contracts in the blockchain system. It takes time to upload the data and proceed with verification. Data that does not meet the preset conditions of smart contracts in the blockchain system will be reprocessed and uploaded for verification. Therefore, for the information transmission of each link in the whole supply chain of grain and oil food, the application of smart contracts can effectively ensure the security of data transmission.

In terms of storage security, since the trusted traceability model of the whole grain and oil food supply chain is designed based on the blockchain architecture, the storage nodes in each link of the supply chain belong to the relevant enterprises or individuals, so the storage of data at each node has certain security risks. However, the artificial modification of the data stored in any link cannot pass the consensus verification of the trusted blockchain system, and any data tampering in each link of the supply chain will be detected. Therefore, its distributed storage architecture ensures the entire supply chain of grain and oil food. The data of each link has high storage security.

**Figure 10:**
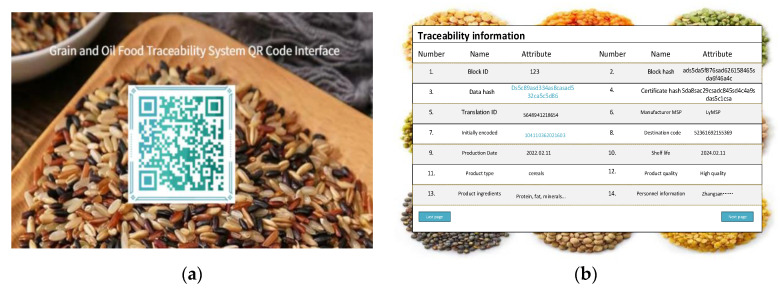
Traceability information query diagram. (**a**) Displays system QR code scanning interface; (**b**) Displays the interface for obtaining detailed data information after scanning is completed.

In order to further ensure the safety performance of this study, this study conducted a safety test on the credible traceability system of the designed grain and oil food supply chain. This paper takes rice as the test object, and conducts traceability queries for rice in a certain period of time in Northeast China. When the QR code of each link is displayed (Figure 10a), the traceability information of the product corresponding to the supply chain link is displayed (Figure 10b).

**Figure 11:**
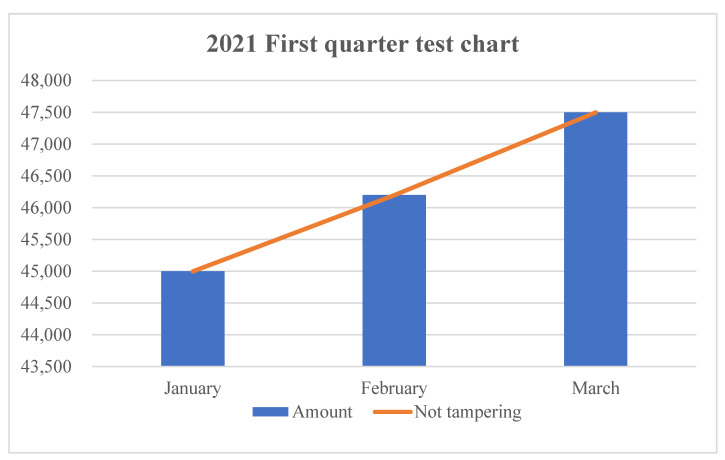
Identification code verification statistics.

There were 1568 batches of rice involved in this time period, and the accuracy rate of traceability information was 100%. This result shows that the system can effectively achieve credible traceability. In addition, in this study, the identification code information verification statistics of a rice processing enterprise are carried out, and the statistical chart is shown in Figure 11. The test time is selected as the first quarter of 2021. Affected by the global new crown epidemic, the cultivation of the processing company has gradually recovered. As can be seen from the figure, the number of tests in the first month is about 45,000 batches, the second month is about 46,200 batches, and the third month is about 47,500 batches, and the number of tampering incidents is almost 0. The results show that the progressive verification of the identification code is carried out in real time by the system, so as to realize the safe transmission of the identification code, so as to ensure the reliable traceability of grain and oil food.

**Figure 12:**
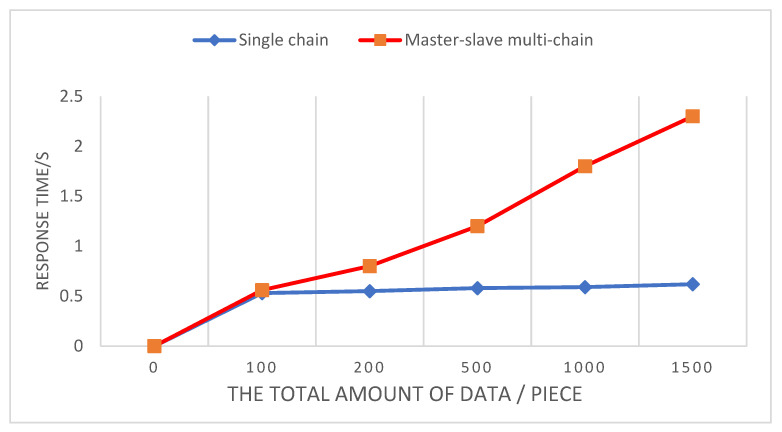
Comparison of query time between single chain and master–slave multi-chain.

**Figure 13:**
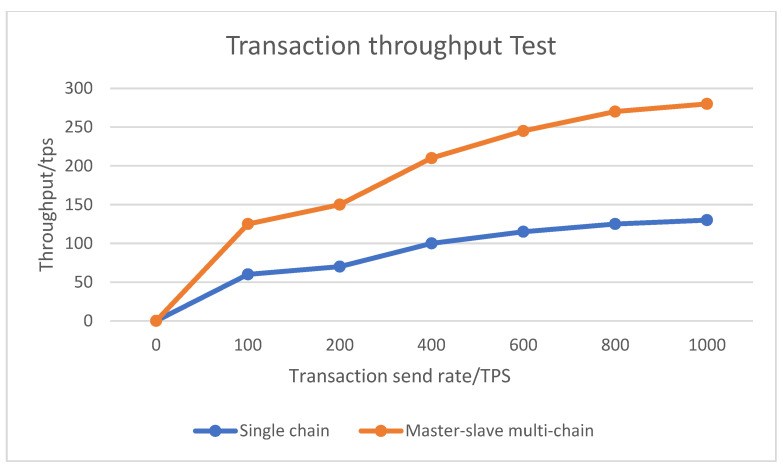
Single-chain and master–slave multi-chain transaction throughput test chart.

In order to verify the operating efficiency of the system, the efficiency and transaction throughput of the single-chain blockchain architecture and the master–slave multi-chain architecture adopted by the system are compared. The total data counts are 100, 200, 500, and 1000. In the case of 1500 records, the time to query 50 records of data at the same time is compared. By comparison, it can be seen that when the amount of data is small, the query time of the two is basically the same, and when the amount of data increases, the single-chain architecture adopts a single consensus mechanism and a large amount of storage, so the query speed increases, while the master–slave multi-chain architecture adopts two different consensus mechanisms, and the amount of data stored on each chain is small, so the query time is basically unchanged. The test results are shown in Figure 12. When the transaction sending rate, that is, the number of concurrent transactions submitted to the system per second, is 100, 200, 400, 600, 800, and 1000, the transaction throughput under the master–slave multi-chain architecture of the blockchain is significantly higher than that of the single blockchain. With regard to the chain architecture, the results are shown in Figure 13.

The analysis shows that with the support of the master–slave multi-chain storage mechanism of the blockchain, the trusted identification and the trusted traceability mechanism, this traceability model can effectively solve the problems of opacity, insecurity and low transaction rate in the whole supply chain of grain and oil food. Reference [6] proposes a research on agricultural product traceability technology (economic value) based on information supervision and cloud computing. In terms of data supervision, he uses cloud storage to operate it, which also gives high quality to the economic value of agricultural product traceability. Compared with Reference [6], this paper adopts the blockchain master–slave multi-chain architecture to trace and supervise data information, and uses the open-source framework Hyperledger Fabric as the development platform, which is more efficient in data security, reliability and economic practicability. In line with sustainable agricultural development, Reference [34] proposes a green blockchain-based traceability platform with low energy consumption and cost savings, suitable for the Fontina PDO cheese supply chain. The traceability system it proposes is based on the Algorand blockchain, which uses pure proof-of-stake consensus, requires minimal computing power, and is highly scalable and environmentally sustainable. Compared with the literature [34], this paper adopts the master–slave multi-chain architecture of blockchain, and the main chain adopts the PoW consensus mechanism, and the slave chain adopts the PBFT consensus mechanism, which has certain advantages in terms of transaction throughput and speed.

## 5. Conclusions and Future Outlook

Based on trusted blockchain and trusted identification, this paper designs a trusted traceability model for the whole supply chain of grain and oil food, which realizes the traceability of information in the whole supply chain of grain and oil food production, processing, warehousing, transportation and sales, which can provide consumers with information. It also can provide consumers or regulatory authorities with specific information on all aspects of the entire product supply chain, and through the application of blockchain and smart contracts, to a certain extent, ensure the safety and reliability of traceability information. At the same time, the trusted identification mechanism designed by this system adopts the identification method combining the new industrial Internet identification and the traditional two-dimensional code, which can effectively improve the timeliness and security of the traceability system. The blockchain master–slave multi-chain storage mechanism adopted by this system can solve the problem that the data and information of the whole grain and oil food supply chain is too large and complex, and the blockchain single-chain architecture can no longer meet its efficiency, and improve the reliability of data information storage. The trusted traceability mechanism provides a safe and reliable traceability solution for the entire grain and oil food supply chain. The overall traceability model can standardize the production process of grain and oil food enterprises and ensure product quality, which is of great significance for solving the problem of grain and oil food safety and the resulting human health and health problems.

At present, most grain and oil food supply chain enterprises have used automated equipment to process all aspects of work, and the supply chain information traceability model formed by the system’s “trusted blockchain + trusted identification” can achieve safe and reliable data and information. However, there are still some small enterprises that use manual input of data information to conduct traceability inquiries, resulting in the inability to guarantee the authenticity of the product quality during the circulation process. Therefore, this has also become a major pain point restricting the implementation of the blockchain traceability system. The harm of false information being authenticated and circulated on the chain can be reduced by means of authoritative centralized agency certification and training of entry personnel through government and other regulatory departments.

Subsequent research will focus on ensuring the authenticity of data collection from the source before the chain, and combine IoT node equipment to automatically collect data to reduce or avoid human intervention, for safety purposes. In addition, future research should try to establish a traceability model that is compatible with different enterprise traceability subsystems, and at the same time improve the modularity of traceability in each link of the supply chain, so as to improve the openness and flexibility of the information traceability model of the whole grain and oil food supply chain.

## Figures and Tables

**Table 1:** Research review table.

Category	Research Content	References
Blockchain-based food or crop traceability solution	Solve the problem of traceability of existing food or crops through blockchain technology	[13,20,22,30,31,32,33,34]
Research on traceability of food or crops based on identification technology	Make the data information of the traceability system credible through identification technology	[23,28,29,35]
Building a traceability solution based on the Internet of Things	Collect and identify information data through IoT devices	[11,36,37,38,39,40]
Agriculture and the Circular Economy	Point out the importance of agriculture for circular economy through research literature	[38,41,42,43]

## Data Availability

Not applicable.

## Appendix A

**Algorithm A1.** SDSC
Input: *H(i)*; *Data*; *D*; *Ds* Authority()//Authorization Template { Verify(*H(i)*)//Authenticate user unique hash } Standardization()//Data Standardization Module { Arrange(*Data*)//The data is matched and arranged to produce D } Summary()//Data table processing module { Hash(*D*)//Get D hash } Storage()//Storage module { Encryption(*Ds*)//digest encryption Chain(*D*,*Ds*)//*D*,*Ds* wind up }

**Algorithm A2.** VDSC
Input: *RDS*; *D* Obtain()//get data module { *D*←RDS//Get CoutchDB’S *D* Hash(*D*)//Get *D* in the CoutchDB database } Judgment()//Judgment module { Decrypt(*Ds*)//Decrypt *Ds* Verify(*Ds*)//Verify *Ds* } Storage()//Storage module { Chain(*R*)//*R* real-time on-chain }

**Algorithm A3.** LRSC
Input: code; *Dc*; *Dd*; Obtain()//Get identification code information module { Get(code)→*Dc*//Get *Dc* } Check()//Check module { Get(*Ds*)//Get *Ds* Verity(*Dc*)//Verify *Dc* } Renew()//Update identification code information module { Addition(*Dd*)//Add the data of this link and update the QR code information } Storage()//Memory module { Transfer(SDSC,VDSC)//Call SDSC,VDSC perform data upload }
